# Charting the Unknown Association of COVID-19 with Thyroid Cancer, Focusing on Differentiated Thyroid Cancer: A Call for Caution

**DOI:** 10.3390/cancers13225785

**Published:** 2021-11-18

**Authors:** Maria V. Deligiorgi, Gerasimos Siasos, Lampros Vakkas, Dimitrios T. Trafalis

**Affiliations:** 1 Clinical Pharmacology Unit–Department of Pharmacology, Faculty of Medicine, National and Kapodistrian University of Athens, 75 Mikras Asias St., 11527 Athens, Greece; smd1700343@uoa.gr (L.V.); dtrafal@med.uoa.gr (D.T.T.); 2 First Department of Cardiology, Hippokration General Hospital of Athens, Faculty of Mediine, National and Kapodistrian University of Athens, 11527 Athens, Greece; gsiasos@med.uoa.gr

**Keywords:** COVID-19, SARS-CoV-2, thyroid cancer, differentiated thyroid cancer, COVID-19 severity, inflammation, immunity, oxidative stress, obesity

## Abstract

**Simple Summary:**

Leveraging lessons learned from the coronavirus disease 2019 (COVID-19) pandemic, resilient health systems focus on the preservation of the continuum of care of chronic diseases, especially of cancer, beyond addressing emergency health requirements. Obesity has a detrimental impact on COVID-19 and affects the epidemic of thyroid cancer (TC). TC, especially differentiated TC (DTC), is a notable paradigm of obesity-related cancers. Thus, obesity–COVID-19–(D)TC interplay can be a constant threat to public health. The present review dissects the COVID-19–(D)TC association in the setting of obesity and beyond, highlighting: (i) the interrelationship between immunity, inflammation, obesity, oxidative stress, and cancer underlying this association; (ii) the challenging management of (D)TC in the COVID-19 era; (iii) the impact of COVID-19 on (D)TC and vice versa; and (iv) the oncogenic potential of SARS-CoV-2. Future perspectives for understanding and harnessing the COVID-19–(D)TC association to inform decision-making are underlined.

**Abstract:**

Background: Conceived of as the “silver lining” of the dark cloud of the coronavirus disease 2019 (COVID-19) pandemic, lessons taught by this catastrophe should be leveraged by medical authorities and policy makers to optimize health care globally. A major lesson is that resilient health systems should absorb sudden shocks incited by overwhelming health emergencies without compromising the continuum of care of chronic diseases, especially of cancer. Methods: The present review dissects the association between COVID-19 and thyroid cancer (TC), especially with differentiated TC (DTC), focusing on available data, knowledge gaps, current challenges, and future perspectives. Results: Obesity has been incriminated in terms of both COVID-19 severity and a rising incidence of TC, especially of DTC. The current conceptualization of the pathophysiological landscape of COVID-19–(D)TC association implicates an interplay between obesity, inflammation, immunity, and oxidative stress. Whether COVID-19 could aggravate the health burden posed by (D)TC or vice versa has yet to be clarified. Improved understanding and harnessing of the pathophysiological landscape of the COVID-19–(D)TC association will empower a mechanism-guided, safe, evidence-based, and risk-stratified management of (D)TC in the COVID-19 era and beyond. Conclusion: A multidisciplinary patient-centered decision-making will ensure high-quality (D)TC care for patients, with or without COVID-19.

## 1. Introduction

Globally, as of the writing of this review more than one and a half years after the emergence of the novel severe acute respiratory syndrome (SARS)-associated coronavirus 2 (SARS-CoV-2), and designation of the attendant coronavirus disease 2019 (COVID-19) as a pandemic, there have been more than 200 million confirmed COVID-19 cases, and more than 4 million related deaths reported by the WHO [1]. This situation, while dismal, has taught us several lessons to be leveraged immediately.

Assessment of the responses of health policies worldwide reveal that the resilience of health systems against health crises of this magnitude lies in going beyond boosting hospital capacity and mitigation of the spread of a virus to maintain continuity of care for all [2]. A major priority of strong health systems is optimization of the care of cancer, which is the second leading cause of death globally [3], with a soaring burden, and which is expected to climb to 28.4 million cases in 2040 (a 47% increase compared to 2020) [4]. 

To forge agile and resilient health systems in the COVID-19 era, it is necessary to reorganize healthcare without compromising the care of cancer [5]. Indeed, the COVID-19–cancer association is bidirectional. On the one hand, cancer care in the COVID-19 era must reconcile conflicting priorities, i.e., mitigation of the risk of SARS-CoV-2 infection and of severe COVID-19 versus prompt cancer diagnosis and treatment despite limited resources and an overwhelmed healthcare system [6]. Delays in diagnosis and treatment, and/or suboptimal treatment of cancer due to the COVID-19 pandemic are clearly acknowledged [4,7,8,9], but quantification will take long time on account of the lag in the dissemination of population-based surveillance data [4]. On the other hand, cancer has been indicated as a prognostic factor for unfavorable outcomes of COVID-19 [10]. 

In that respect, a critical mass of scientists has committed to transcending the boundaries of faculties to untangle the COVID-19–cancer association and address emerging challenges in a multidisciplinary way. Central to this initiative is the association of COVID-19 with cancers related to obesity, which is defined as a body mass index (BMI) ≥30 kg/m^2^ [11]. The rationale for this concept is that obesity—designated as an epidemic by the WHO in 1998 [11]—is the main culprit of the global burden of cancer [12] and of unfavorable outcomes of COVID-19 [13,14]. Additionally, obesity has been suggested as a critical player in the pathophysiological background of the COVID-19–cancer association [15]. 

Thyroid cancer (TC)—especially differentiated thyroid cancer (DTC), which arises from thyroid follicular epithelial cells, and accounting for the vast majority of TC [16]—is the most common endocrine malignancy [17]. Despite its indolent nature [17], TC poses a rising burden on public health, and is attributed mostly to obesity [18,19,20,21,22]. In fact, TC can be considered as the epitome of obesity-related cancers.

Although thyroid is a well-known target of SARS-CoV-2 [23], the COVID-19–TC association remains terra incognita; however, the COVID-19–TC association is of paramount clinical importance in the context of obesity, and beyond, for many reasons. First, the mounting burden of obesity promotes both an explosion of TC incidence and unfavorable outcomes of COVID-19. Second, obesity may contribute to unfavorable outcome COVID-19 in TC patients. Third, TC provides a prism for investigating the pathophysiological background of the COVID-19–cancer association. Fourth, the oncological strategy for management of TC has been reformed in the COVID-19 era. Finally, TC may worsen COVID-19 outcomes. 

Prompted by this, the present review dissects COVID-19–TC association in the context of obesity, and beyond, with a focus on knowledge gaps, current challenges, and future perspectives.

Most available data concerning the association of COVID-19 with TC in the context of obesity and beyond provide no information about distinct histological TC types, or exclusively concern DTC. Herein, this approach is represented by the term “COVID-19–(D)TC association”.

## 2. TC Epitomizes the Obesity-Related Cancers

With 586,202 new cases globally in 2020, TC ranks as the 9th most common cancer type. Although TC most often shows low mortality rates (0.5 per 100,000 in women and 0.3 per 100,000 in men) [17], it constitutes a growing healthcare challenge due to rising incidence, and is nominated as a “TC epidemic”. This extends beyond an “epidemic of diagnosis” to reflect the increasing exposure of populations to risk factors, among which obesity prevails [18,19,20,21,22]. 

TC is included among the 13 cancer types causally associated with obesity, according to the landmark report by the International Agency for Research on Cancer (IARC) [24]. The causality between excess body weight and obesity-related cancers is sustained by accumulating epidemiological and experimental evidence [11,25,26,27]. A convincing argument favoring the contribution of obesity to cancer risk is the recognition of a decrease in the risk of certain cancer types as a collateral beneficial effect of bariatric surgery [28].

It is acknowledged that obesity has a differential impact on distinct TC types [24]. DTC, which comprises more than 90% of all thyroid malignancies, including papillary thyroid carcinoma (PTC) (the most common histotype) and follicular thyroid carcinoma (FTC) (the 2nd most-common histotype), is the histological TC type that is most often positively associated with obesity. Medullary thyroid cancer (MTC), accounting for less than 5% of all TC types, is a neuroendocrine tumor with a distinct profile [29] and has an inverse association with obesity [24]. Anaplastic TC is another obesity-related TC type [24], but it is rare, accounting for 1.7% of TC in the United States, and for 1.3% to 9.8% (median = 3.6%) of TC geographically [30].

A hallmark systematic analysis of data from the Global Burden of Disease (GBD) 2017 study of 195 countries and territories showed that 11.8% of worldwide TC-related deaths in females was attributable to high a BMI; a percentage that was higher compared to that in males. Interestingly, the highest proportion of TC-related deaths attributable to high BMI occurred in developed countries and in middle-aged individuals [18]. 

Analysis of the influence of obesity on PTC incidence trends in the US during the period of 1995–2015 showed that, compared to normal weights (18.5–24.9 kg/m^2^), overweight (25.0–29.0 kg/m^2^) and obesity (≥30.0 kg/m^2^) increased the risk of PTC by 1.26-fold (95% confidence interval (CI), 1.05- to 1.52-fold) and 1.30-fold (95% CI, 1.05- to 1.62-fold), respectively, as well as the risk of large (>4 cm) PTC (hazard ratio (HR), 2.93; 95% CI, 1.25–6.87 for overweight; and HR, 5.42; 95% CI, 2.24–13.1 for obesity). Over 1995–2015, an increase in population-attributable fractions (PAF) for overweight and obesity was observed, from 11.4% to 16.2%, for all PTCs, and from 51.4% to 63.2% for large PTCs. Overweight and obesity were responsible for 13.6% and 57.8% of the annual percent changes in all (5.9%/year) and large (4.5%/year) PTC incidences, respectively [22].

There is ample epidemiological evidence sustaining the tumor-promoting role of obesity in TC [21,24,28,31,32]. The underlying mechanisms have not been fully elucidated, but they implicate five different players: thyroid-stimulating hormone (TSH) [28], estrogens [28,33], insulin resistance (IR) [28,31], inflammation [28], and adipokines [28,31]. 

Adipokines—a subset of cytokines produced by adipose tissue—are considered the orchestrators of the interrelationship between obesity, IR, and inflammation. The main alterations in the secretion of adipokines, which are closely related to the obesity–TC association, are a decrease of adiponectin (APN), an increase of leptin, and an increase of resistin.

A critical link between obesity and TC is a decrease in APN levels, which undermines the tumor-suppressive role of APN [31,34]. Downstream of decreased APN levels are promoted signaling pathways that counteract the APN-mediated tumor-suppressive signaling pathways, fostering tumor initiation and progression through (1) inhibition of the adenosine monophosphate-activated protein kinase (AMPK), which in turn allows the activation of both mitogen-activated protein kinase (MAPK) and phosphoinositide-3 kinase (PI3K)/protein kinase B (PKB) (Akt)/mammalian target of rapamycin (mTOR) pathways, eventually favoring cancer cell proliferation; (2) downregulation of the expression of important factors involved in the arrest of the cell cycle and apoptosis, such as p21 and p53; (3) inflammatory and immunomodulating tumor promoting effects; (4) pro-angiogenetic effects; (5) a decrease in the APN/leptin ratio, favoring the action of excess fat-driven increase of leptin, which exerts a tumor-promoting role mediated by Akt/mTOR/PI3K, extracellular signal-regulated kinases (ERK)/MAPK, and Janus kinase (JAK)/signal transducer and activator of transcription 3 (STAT3) cascades; and (6) the unopposed carcinogenic effect of chronic hyperinsulinemia. The carcinogenic effect of chronic hyperinsulinemia is attributed to (i) stimulation of MAPK and PI3K/Akt pathways; (ii) an increase in the bioavailability and synthesis of insulin-like growth factor 1 (IGF-1); and (iii) overexpression of vascular endothelial growth factor (VEGF), favoring pro-angiogenic pathways [28,31,34,35,36].

Increased levels of resistin produced by excess body fat can stimulate cancer cell proliferation and survival, as well as pro-inflammatory and pro-angiogenic pathways, which are critical for the promotion of TC [28].

**Figure 1:**
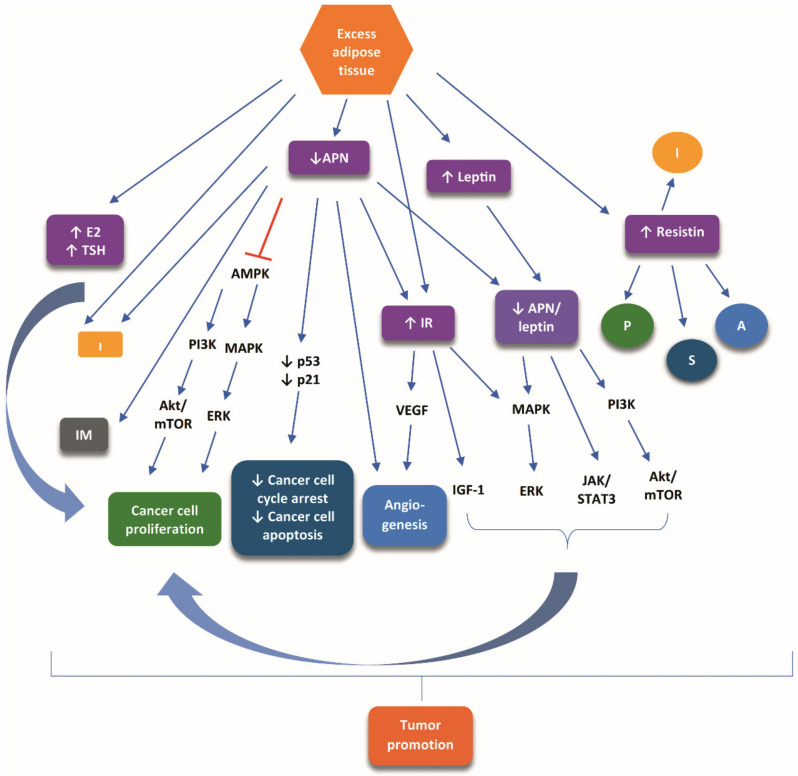
The main signaling pathways presumed to mediate the tumor-promoting role of obesity in TC. Excess adipose tissue causes an increase in TSH and 17-β-estradiol, decrease of adiponectin, insulin resistance, and increase of leptin and resistin. These alterations modulate numerous signaling cascades, which crosstalk and result in tumor promotion. Symbols: increase: ↑; inhibiton: 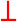; stimulation: 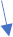. Abbreviations: A, angiogenesis; Akt, protein kinase B; AMPK, adenosine monophosphate-activated protein kinase; APN, adiponectin; E2, 17-β-estradiol; ERK, extracellular signal-regulated kinases; I, inflammation; IGF-1, insulin-like growth factor 1; IM, inflammatory-immune responses; IR, insulin resistance; JAK, MAPK; mitogen-activated protein kinase; mTOR, mammalian target of rapamycin; P, proliferation; PI3K, phosphoinositide-3 kinase; S, survival; STAT3, signal transducer and activator of transcription 3; TSH, thyroid-stimulating hormone; VEGF, vascular endothelial growth factor.

Figure 1 illustrates the main signaling pathways presumed to mediate the tumor-promoting role of obesity in TC. 

The association of obesity with TC is not a consistent finding. Analysis of a retrospective series of 4849 patients with thyroid nodules by Rotondi et al. did not confirm an association between obesity and DTC [37]. Furthermore, analyses from two national data sources—the military recruitment health examinations and the Israel National Cancer Register—totaling 24,389,502 person-years of follow-up, by Farfel et al., revealed 60 incidence cases of TC, but no association between BMI and TC incidence [38]. Additionally, Fussey et al. analyzed data from 1812 participants with benign thyroid nodular disease and 425 with DTCs from the UK Biobank, and demonstrated that higher BMI was positively associated with benign thyroid nodular disease (odds ratio (OR), 1.15; 95% CI, 1.08–1.22) and higher waist-hip ratio (OR, 1.16; 95% CI, 1.09–1.23), but not with TC. Mendelian randomization revealed no causal association between obesity and benign nodular thyroid disease or TC, but an increased risk of TC in the highest quartile of the genetic liability of type 2 diabetes mellitus (DM 2) compared to the lowest quartile (OR, 1.45; CI, 1.11–1.90) [39]. No interpretation of the discordant evidence on the contribution of obesity to TC exists thus far. Table 1 summarizes controversial data on the association between obesity and (D)TC.

## 3. Obesity in Relation to the Severity of COVID-19 in Non-Cancer and in Cancer Patients

In the general population, obesity is widely acknowledged as a bona fide risk factor for increased severity of COVID-19, resulting in higher rates of intensive care unit (ICU) admission, mechanical ventilation, and mortality compared to non-obesity [12,13]. This effect can be partially explained by the recognition of chronic non-communicable diseases (NCDs) related to obesity, e.g., DM 2 [40], hypercholesterolemia [41], hypertension, and cardiovascular diseases [42], as risk factors for increased severity of COVID-19. However, obesity has been proven an independent significant factor for unfavorable COVID-19 outcome after adjusting for coexistent NCDs. In fact, several mechanisms increasing the severity of COVID-19 in obese patients have been suggested; a detailed presentation of this is beyond the scope of the present review. First, excess fat can trigger many metabolic disorders, i.e., aberrant fatty acid metabolism, cellular hypertrophy and death, endoplasmic reticulum (ER) stress, hypoxia, and mitochondrial dysfunction, forging a pro-inflammatory milieu that is both local and systemic, which, in turn, can attenuate the immune response. Second, the immune response can be compromised by IR and leptin resistance attributed to excess fat. Third, up-regulation of the expression of angiotensin-converting enzyme 2 (ACE-2) in adipose tissue may cause adipose tissue to be a deposit reservoir of SARS-CoV-2 in the host, given that ACE-2 is co-opted by SARS-CoV-2 in order to enter host cells. Fourth, obesity is correlated with a hypercoagulable state, marked by elevated levels of prothrombin factors and reduced levels of anti-thrombin molecules, which can aggravate COVID-19-related coagulopathy/thrombosis [43,44,45,46,47]. Finally, obese COVID-19 patients may have increased abdominal pressure, limited chest expansion and movement, and insufficient respiratory compensatory function, parameters that can easily drive lung infection to respiratory failure [48]. 

However, whether obesity is a determinant of increased severity of COVID-19 in cancer patients remains uncertain. A single-center, prospective cohort study conducted in France, enrolling 178 mixed ambulatory and hospitalized patients with cancer, showed that obesity was not a risk factor for clinical worsening and overall survival (HR, 1.16; 95% CI, 0.36–3.69) in a univariable Cox proportional model [49].

A cohort study of 928 patients with COVID-19 and cancer revealed no association between obesity and increased 30-day all-cause mortality (OR, 0.84; 95% CI, 0.50–1.41), even after adjusting for age, sex, and smoking status (OR, 0.99; 95% CI, 0.58–1.71) [50].

A systematic review/meta-analysis of obesity and all-cause mortality in cancer patients with COVID-19 retrieved 3387 studies, and only three retrospective cohort studies reported outcomes according to obesity status. These three studies were multi-national and enrolled 419 obese and 1694 non-obese cancer patients with COVID-19 in both inpatient/outpatient settings. Analysis of these three studies showed no correlation between obesity and all-cause mortality (OR, 0.95; 95% CI, 0.74–1.23) in cancer patients with COVID-19. Heterogeneity was low (I^2^ = 33%) and no significant funnel plot asymmetry was identified using Egger’s test (*p* = 0.2273) [51]. 

The absence of an association of obesity with COVID-19 severity and mortality in cancer patients is difficult to interpret. This notion seems reminiscent of the so-called “obesity paradox”, i.e., the association of obesity with a favorable cancer prognosis [46,52]. The “obesity paradox” is observed, not only in head and neck cancers, but also in premenopausal breast cancer, non-small cell lung cancer (NSCLC), renal cell cancer (RCC), and metastatic colorectal cancer (CRC) [52]. Avgerinos et al. suggested that the “obesity paradox” in cancer patients is ascribed, at least partially, to methodologic issues, such as: (i) selection, stratification, and detection biases; (ii) application of BMI as an index of general adiposity; and (iii) confounding factors (age, smoking, physical activity, etc.) [52]. On the other hand, Park et al. assumed that the “obesity paradox” insinuates a greater metabolic reserve favored by abundant adipose tissue, enhancing patient resilience [46]. In that respect, the absence of an association between obesity and COVID-19 severity and mortality in cancer patients may reflect methodological issues or insinuate an enhanced resilience of obese cancer patients against COVID-19 compared to non-obese cancer patients. More studies are needed to confirm or refute this hypothesis. Table 2 summarizes the discordant data regarding the contribution of obesity to COVID-19 outcomes in non-cancer and cancer patients. 

## 4. Pursuing the Pathophysiological Landscape of the COVID 19–TC Association

Thus far, the pathophysiological landscape of the COVID-19–cancer association remains elusive. However, an insightful hypothesis was proposed by Derosa et al. Based on accumulating datasets, Derosa et al. assumed that cancer and COVID-19 meet at the crossroad of inflammaging—a state of aberrant tuning of systemic inflammation due to cytokine dysregulation ascribed to remodeling of immune system—and immunosenescence—a state of diminishing functioning of the immune system. Both states are promoted by obesity and risk factors for diminished immunity and stimulation of inflammation (e.g., aging), as well as cancer and poor performance status [15]. 

In fact, immunosenescence and inflammaging can compromise innate and adaptive immune responses, aggravating both overt inflammation and cancer dissemination, predisposing to SARS-CoV-2 infection, increasing the risk of severe COVID-19, and favoring cancer progression. Additionally, obesity can promote cancer initiation and progression through contribution to the acquisition of all hallmarks of cancer [14], increased insulin resistance resulting in hyperinsulinemia, and altered adipokines. Cancer per se, especially advanced and metastatic cancer, may subvert the fine balance between viral replication and appropriate innate immune responses (for example, type I and III interferon (IFN)), as well as cognate immune responses (for example, memory T helper 1 (Th1) response and antibody-secreting cell cross-reactivity with other beta coronaviruses), favoring virus replication [15].

Furthermore, chemotherapy and radiotherapy can also cause deficits in immune response, interrelated with and critical for long-term protective anticancer and antiviral immune responses, e.g., type I and II IFN responses. Such deficits may favor, not only cancer progression, but also vulnerability to viral infections [15]. Furthermore, major adverse effects of conventional therapies (such as cytotoxic chemotherapy, hormone therapy, and radiotherapy), targeted therapies (such as tyrosine kinase inhibitors (TKI) and mTOR inhibitors) and immunotherapies (such as immune-checkpoint inhibitors and CAR T cells), as well as poor patient performance status, can aggravate the outcome of COVID-19. 

Alarmingly, comorbidities that act as trigger points for the COVID-19–cancer association (e.g., obesity, aging) may also undermine the efficacy of immune-based anticancer and antiviral therapies [15]. 

Another presumptive link between COVID-19 and cancer is oxidative stress—a key player in both COVID-19 [53,54] and cancer [55,56]. Oxidative stress is defined as a relative excess of chemically reactive oxygen species (ROS) compared to antioxidants [55]. Superoxide, the precursor of most ROS, is formed by the univalent reduction of triplet-state molecular oxygen (^3^O_2_), enzymatically (e.g., by NAD(P)H oxidases) or non-enzymatically. ROS stem from endogenous sources (e.g., mitochondria, peroxisomes, lipoxygenases, NADPH oxidases, cytochrome P450), antioxidant defense systems (e.g., enzymatic, and non-enzymatic systems), or exogenous sources (ultraviolet light, ionizing radiation, chemotherapy, and environmental toxins) [57,58]. Although low/moderate levels of ROS are critical for various physiological functions, increased ROS levels undermine genome stability and cellular integrity, contributing to aging and cancer [58,59,60], including TC [61]. SARS-CoV-2 may promote oxidative stress via downregulation of ACE2, the hallmark protein co-opted by SARS-CoV-2 for its internalization. ACE2 is a key regulator of the renin–angiotensin–aldosterone system (RAS), promoting the conversion of angiotensin II into angiotensin-1,7. The latter is credited with counteracting the negative effects of RAS, exerting anti-hypertensive, anti-inflammatory, anti-fibrotic, and anti-oxidative effects, which are protective for the heart, blood vessels, kidney, and central nervous system. It has been shown that SARS-CoV-2 not only binds to ACE2 via its spike protein (S) to enter host cells, but it can also downregulate ACE2 expression in cells, thereby disrupting the protective effects of angiotensin 1–7 [62]. Accumulating data suggest a key role for oxidative stress in the pathogenesis and severity of COVID-19 [53]. 

**Figure 2:**
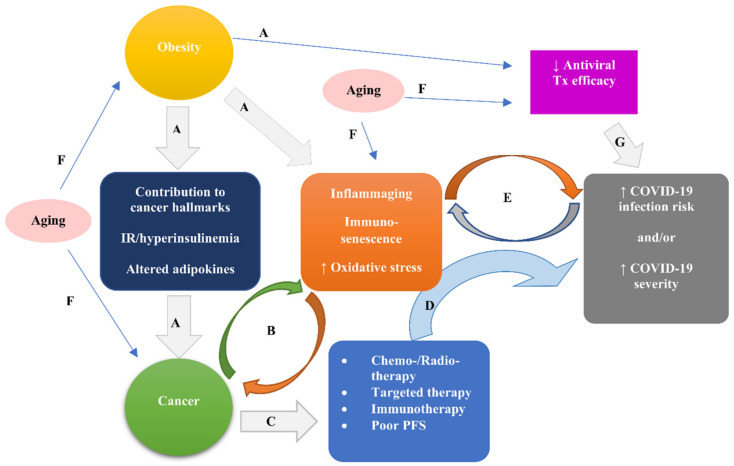
The interplay between obesity, cancer, immunity, and oxidative stress presumed to underlie the pathophysiological landscape of the COVID 19–cancer association. Obesity via contribution to all cancer hallmarks, and stimulation of insulin resistance leading to hyperinsulinemia, and of altered adipokines can promote cancer initiation and progression (A). The obesity-elicited states of inflammaging, immunosenescence, and increased oxidative stress (A) can promote and be promoted by cancer (B). Cancer due to attendant treatments (i.e., chemo-/radio-therapy, targeted therapy, and immunotherapy) and possible poor performance status (C) can increase susceptibility to and severity of COVID-19 (D). COVID-19 risk and/or severity can aggravate and be aggravated by the states of inflammaging, immunosenescence, and increased oxidative stress (E), the latter being elicited by obesity (A) and/or cancer (B). Aging can be a risk factor for obesity, cancer, inflammaging, immunosenescence, and increased oxidative stress (F), leading eventually to increased risk and/or severity of COVID-19 via mechanisms A, B, C, D, and E. Additionally, both aging (F) and obesity (A) may decrease the efficacy of immune-based antiviral treatments, increasing the severity of COVID-19 (G). Abbreviations: COVID-19, coronavirus disease 19; PFS, performance status; Tx, treatment.

Figure 2 illustrates the interplay between obesity, cancer, immunity, and oxidative stress presumed to underlie the pathophysiological landscape of COVID 19–cancer association. 

The COVID-19–(D)TC association provides a prism for understanding COVID-19–cancer association in many aspects. First, the key host proteins co-opted by SARS-CoV-2 to invade host cells, i.e., ACE2 and transmembrane protease serine 2 (TMPRSS2), are amply expressed in the thyroid [63]. Second, internalization of SARS-CoV-2 in the host’s target cells is mediated by integrin αvβ3 [64], which mediates the non-genomic actions of thyroid hormones, especially of those of thyroxine (T4). Accordingly, T4 binding to integrin αvβ3 may influence the internalization of SARS-CoV-2. Third, the hypothalamus–pituitary–thyroid axis is a well-documented target of SARS-CoV-2 [65], and of the related cytokine storm, resulting in a wide spectrum alterations to thyroid function [66]. Fourth, the long-pursued, yet not clarified, inflammatory and immunological background of DTC, discussed by two of the authors of this review (M.V.D, D.T.T) elsewhere [67], can interact with the immune-inflammatory reactions triggered by SARS-CoV-2 [68]. Fifth, there are convincing data sustaining the critical role of oxidative stress—a key player of COVID-19—in DTC, namely: (i) the exposure of follicular thyroid cells to a great amount of H_2_O_2_ (a landmark member of non-radical ROS), which is essential for synthesis of thyroid hormones; (ii) the detection of oxidative stress and DNA damage across the continuum of thyroid carcinogenesis from thyroid adenomas to advanced stages of thyroid cancer; and (iii) the upregulation of two main ROS-generating systems, the NADPH oxidases DUOX1 and NOX4, by ionizing radiation and mutated oncogenes RAS and BRAF—the most well-described carcinogenic factors for DTC [62]. Most importantly, obesity is a common risk factor shared by TC and COVID-19.

Albeit speculative, the conceptualization of the pathophysiological landscape of the COVID 19–TC association discussed herein suggests many actionable pathways, which merit further evaluation.

## 5. The Impact of COVID-19 on the Oncological Strategy for DTC

In the face of the reorganization of health systems compelled by the COVID-19 pandemic, the oncological strategy for the management of DTC raises many challenges.

### 5.1. Challenge I: Appropriate Patient Selection for Thyroid Fine-Needle Aspiration (FNA)

Thyroid fine-needle aspiration (FNA) is the cornerstone of the diagnostic procedure of DTC [16]. Appropriate patient selection for FNA of thyroid nodules is based on clinical and ultrasound (US) findings, and constitutes the first step in decision-making concerning the diagnosis and the management of DTC. This step entails constructive crosstalk between endocrinologists and cytopathologists [16]. In general, proper requests of thyroid FNA by endocrinologists, justified by clinical and US features of thyroid nodules suspicious of malignancy, increase the diagnostic efficiency of FNA. On the contrary, improper requests of thyroid FNA undermine the diagnostic efficiency of FNA. In the COVID-19 era, the recommended, due to the pandemic, delay of all non-urgent diagnostic procedures, including thyroid FNA, renders the interaction of endocrinologists and cytopathologists more important than ever. 

Indeed, analysis of the thyroid FNA trends at Federico II University of Naples before (1 January 2019 to 13 March 2020), during (14 March to 15 May), and after (16 May to 7 July) the first lockdown in Italy revealed a decrease in the average weekly number (AWN) of FNA, from 62.1 to 23.1. In fact, the weekly proportion of benign diagnoses decreased on average by 12%, while the weekly proportion of high-risk diagnoses increased by 6%. This study sustains that the lockdown urged referring endocrinologists to prioritize thyroid FNA for nodules with US and/or clinical features indicative of high-risk of malignancy [69]. 

Consistent with this finding, an international survey of cytopathologic laboratories in 23 countries assessing the global impact of the COVID-19 pandemic on cytopathology practice revealed that, overall, the sample volumes collected during four weeks of COVID-19 lockdown were lower compared to those obtained during the corresponding period in 2019 (104,319 samples versus 190,225 samples, respectively), with an average volume decrease of 45.3% (range, 0.1–98.0%). The percentages of samples were either increased or decreased depending on the biopsied organ. The percentage of thyroid samples was significantly decreased, from 5.02% to 3.26% (*p* < 0.001). However, the overall decrease in thyroid FNA cytology volume was less profound for samples that usually reveal a higher rate of malignancy, thanks to appropriate patient selection for thyroid FNA [70].

Analyses of databases from China, South Korea, Iran, and Italy during COVID-19 pandemic phase I (25 January–25 February 2020), phase II (26 February–19 March), and phase III (20 March–20 April), compared to corresponding periods of 2019, revealed a reduction in outpatient FNA by 99.7% in phase I, 62.9% in phase II, and 30.1% in phase III [71]. 

### 5.2. Challenge II: Schedule of DTC Surgery in an Overburdened Health System

In view of the urgent need to mitigate the risk of transmission of SARS-CoV-2 and optimize the allocation of limited hospital beds and financial resources, healthcare providers are forced to prioritize the treatment of COVID-19 patients and emergency cases, while postponing elective surgical procedures of all types. 

Assessment of global real-world data demonstrated the cancellation of more than 28 million surgeries in the face of the COVID-19 pandemic in March 2020. Data collected through interviews with surgical experts from 190 countries demonstrated that approximately 72% of non-essential surgeries were cancelled, i.e., a total of 28,404,603 surgeries globally. Among the cancelled surgeries, most cases concerned benign pathology (90%), while only 8.2% concerned tumor [72]. 

DTC surgeries are among the most common elective operations in clinical practice. Nevertheless, given the uncertain end of COVID-19 pandemic, the long-term delay of essential thyroid surgical care can be more detrimental than COVID-19 per se. Confronted with this challenge, several distinct panels of experts recommend alternative treatment algorithms as tangible options for the management of thyroid tumors with a focus on DTC for patients who cannot be operated on under the current circumstances. All expert opinions highlight that COVID-19 guidelines for the management of DTC are not applicable under circumstances where surgical treatment is feasible.

COVID-19 pandemic guidelines suggested by a panel of 67 experts (45 endocrine surgeons and 22 endocrinologists) from a wide array of countries recommend a modified risk-stratified approach to thyroid surgery. Postponement of surgery for 3–6 months and alternative treatment options are recommended for patients who are candidate for surgery due to a twice-confirmed result of FNA indicative of atypia/follicular lesion of undetermined significance (AUS/FLUS) (Bethesda 3), or Hürthle cell neoplasia/follicular neoplasia (Bethesda 4), or papillary microcarcinoma without pathological lymph nodes in the neck, or carcinoma without pathological lymph nodes in the neck. Such alternative treatment options are minimally invasive ablation techniques, including laser, microwave, or radiofrequency ablation, and active surveillance until the end of the pandemic [73].

COVID-19 pandemic guidelines suggested by a panel of experts-members of the French-speaking Association of Endocrine Surgery (AFCE) recommend that, in the absence of signs of locoregional aggressiveness of DTC, delay of thyroid surgery should be tailored according to tumor size and lymph node status. Especially, surgery of tumors ≥2 cm with or without lymph node metastases can be postponed until the end of crisis, but then, it should be prioritized with a delay shorter than 3 months. Surgery of tumors <2 cm can be performed after the end of COVID-19 pandemic [74].

COVID-19 pandemic guidelines from the Turkish Association of Endocrine Surgery recommend that indeterminate nodules (classified according to cytopathologic findings of FNA as Bethesda 3 and 4 categories) can be subjected to active surveillance, while the positivity of molecular markers (e.g., BRAF, TERT) does not impose immediate surgical intervention. In case of malignant nodules (classified according to cytopathologic findings of FNA as Bethesda 4 and 5 cytopathologic categories), management is guided by risk stratification. DTC with low and intermediate risk of recurrence according to American Thyroid Association (ATA) classification, and intrathyroidal papillary microcarcinomas (PTMC) can remain under active surveillance if US examination is conducted periodically. In the case of DTC with a high risk of recurrence according to ATA classification, i.e., large compressive or locoregionally aggressive DTC, thyroid surgery should be performed immediately. Cross-sectional imaging (computed tomography (CT), magnetic resonance imaging (MRI)) may help to determine the extension of thyroid surgery [75].

A position statement from the thyroid department of the Brazilian Society of Endocrinology and Metabolism (SBEM) highlighted that most TC cases are at low risk of recurrence, associated with an excellent prognosis, and thus “surgical procedures could and should be postponed safely during the pandemic period”. Additionally, this position statement endorsed the possibility to safely postpone radioiodine (RAI) therapy in case or a pertinent indication [76]. All COVID-19 guidelines for the management of thyroid tumors with focus on DTC are depicted in Table 3.

Real-world data on the DTC surgery during the COVID-19 pandemic are limited. Analysis of databases from China, South Korea, Iran, and Italy concerning thyroid surgery during COVID-19 pandemic phase I (25 January–25 February 2020), phase II (26 February–19 March), and phase III (20 March–20 April) compared to corresponding periods of 2019 revealed that no thyroid surgeries were performed during phase I. A reduction in surgery of advanced DTC was observed in phase II—concerning DTC of stage T1bN1a—and in phase III—concerning DTC of stage T3bN1b [71]. DTC of stage T1b N1a is between 1 cm and 2 cm completely inside the thyroid (T1b) and has spread to pretracheal, paratracheal, and prelaryngeal lymph nodes (level VI), or to the superior mediastinal nodes (level VII) (N1a). DTC of stage T3bN1b is of any size with extrathyroid extension to one or more of the muscles beside the thyroid (strap muscles) (T3b), and to lateral lymph nodes (levels I, II, III, IV, V) or to retropharyngeal lymph nodes (N1b) [16]. Mean surgical time was reduced to 58.3 ± 11.26 min (*p =*  0.000) in phase II versus 2019 and to 55.3 ± 13.32 min in phase III versus 2019 (*p =*  0.000). Postoperative hospitalization was reduced to 2.8 ± 0.9 days (*p =*  0.000) in phase II versus 2019, and to 3.3 ± 1.0 days (*p =*  0.008) in phase III versus 2019 [71].

### 5.3. Challenge III: Assessment of Delays and Disruptions in DTC Care Due to COVID-19

To assess delays and disruptions in DTC care due to COVID-19, standardizing the measurement and reporting thereof is required. 

A systematic review of 62 studies, wherein head and neck cancer patients represented 11% of enrolled patients, identified interruptions in patient care in terms of facilities (up to 77.5%), supply chain, including everything from drugs to technical support of imaging equipment (up to 79%), and availability (up to 60%). The most common delays or disruptions were reductions in routine activity of cancer services and number of cancer surgeries, delay in radiotherapy, and delay, reschedule, or cancellation of outpatient visits. The result was delay in treatment, diagnosis, or general health service [77]. 

To date, to the best of our knowledge, large studies addressing this issue in the context of DTC are still lacking. A small relevant retrospective study evaluated the impact of lockdown and restrictive policies in Jordan between 17 March and 20 May 2020, on PTC treatment plans for 12 PTC patients with an average tumor size of 44 mm (range: occult–80 mm). Patients were subjected to thyroid surgery for initial management of PTC (11 patients), or recurrence of PTC (1 patient), without any delay of planned surgery. However, the lockdown across international borders decreased the availability of RAI. Consequently, in cases where RAI was indicated, replacing the usual method for the delivery of RAI after levothyroxine (L-T4) withdrawal with the recombinant human (rh) TSH (rh-TSH)-stimulated method in most patients was required. However, given limited financial resources, patients who were unable to afford the additional cost for the rh-TSH-stimulated method experienced increased stress and were classified to mild-to-moderate anxiety group according to the Hamilton Anxiety rating scale (HAM-A scale) [78]. With the increasing number of studies addressing delays in cancer care due to COVID-19, it would be of interest to separately analyze the relevant data regarding DTC to facilitate the design of policies to mitigate this phenomenon.

### 5.4. Challenge IV: The Safety of DTC Surgery in the COVID-19 Era

The COVID-19 pandemic has created novel barriers to safe head and neck surgery due to intense SARS-CoV-2 replication in the upper aerodigestive tract and aerosol generating head and neck surgical procedures. Thus, a systematic review on head and neck practice during the COVID-19 pandemic inferred that the cancer should be treated in compliance with specific protection measures for medical staff and specific guidelines for patient management [79]. 

Fortunately, a systematic review on thyroid surgery during COVID-19 pandemic revealed a relatively low risk of SARS-CoV-2 transmission during thyroid surgery. This review analyzed data from nine studies performed across several countries between January and August 2020, in countries with SARS-CoV-2 prevalence in the community varying from low to high. A total of 2217 patients who underwent thyroid surgery were enrolled. Malignancy was the indication for surgery in 60.8% of patients, 83.4% of whom were subjected to total thyroidectomy, accompanied by lymph node dissection in 38.3% of cases. Cross-infections were reported in 14 out of 721 patients (1.9%), while severe pulmonary complications of COVID-19 occurred in 0.4% of infected patients. Based on four studies, the incidence of complications related to thyroid surgery was 20.2%, with hypoparathyroidism and recurrent laryngeal nerve injury being the most common [80]. 

The prospective database of the Division of Thyroid Surgery in China-Japan Union Hospital of Jilin University of Changchun searched for patients who underwent thyroid surgery during phases I, II, and III of COVID-19 pandemic in China, South Korea, Iran, and Italy revealed no cases of COVID-19-related adverse events during the perioperative period [71].

An international, observational cohort study of 1137 consecutive head and neck cancer patients who underwent primary surgery in 26 countries indicated the thyroid as the second most common site of surgery (21%) after the oral cavity (38%). The overall 30-day mortality was 1.2%, similar to pre-COVID data; however, percentage of positivity of SARS-CoV-2 tests within 30 days of surgery was the same in patients and in the surgical team (3%, each), pointing to ineffective cross-infection measures or personal protective equipment. Among patients infected by SARS-CoV-2, 44.8% experienced severe respiratory complications, and 10.3% died. Medical and surgical complications were within ranges normally seen for the whole cohort. Advanced tumor stage was significantly associated with admission to critical care. Taken together, the authors highlighted that head and neck cancer surgery in the COVID-19 era is safe, but this fact does not undermine the need for compliance with safety measures [81]. 

The challenges in the oncological strategy for DTC due to the COVID-19 pandemic and relevant studies are depicted in Table 4.

**Figure 3:**
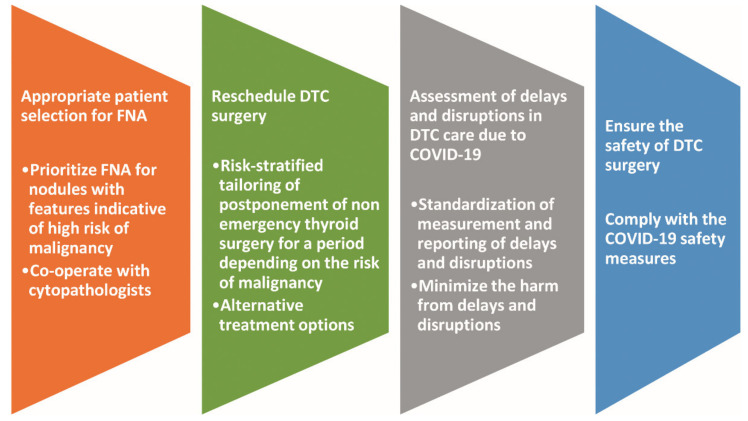
Challenges in the oncological strategy for DTC due to COVID-19 pandemic and the corresponding counteracting initiatives. Abbreviations: COVID-19, coronavirus disease 19; DTC, differentiated thyroid carcinoma; FNA, fine needle aspiration.

Figure 3 illustrates the challenges in the oncological strategy for DTC due to the COVID-19 pandemic and the corresponding counteracting initiatives. 

## 6. Discordant Data on the Impact of TC on COVID-19 Severity between Cancer Patients in General and (D)TC Patients

The first estimation of the probability of COVID-19 related death in a large sample of cancer patients with COVID-19 came from China and showed that the COVID-19-related mortality rate in cancer patients was 28.6%, more than ten times higher than that reported in all COVID-19 patients in China [82]. Thus far, it has been constantly reported that cancer patients are more vulnerable to COVID-19 and to unfavorable outcomes of COVID-19 compared to patients without cancer [50,83,84,85]. Metastatic lung cancer and hematological cancer confer the greatest risk of severe complications of COVID-19 and related mortality [86]. The interrelationship between SARS-CoV-2 and cancer has been reviewed by van Dam et al. [87] and is beyond the scope of the present review. Herein, we discuss some representative data.

The largest study to date addressing the impact of cancer on COVID-19 risk and outcome is a retrospective case-control analysis of patient electronic health records enrolling 73.4 million patients from 360 hospitals and 317,000 clinicians across the 50 US states. Evaluation of the odds ratio of COVID-19 infections for 13 common cancer types revealed that cancer patients before and after adjusting for COVID-19 risk factors had significantly increased risk for COVID-19 infection compared to patients without cancer after adjusting for COVID-19 risk factors.

The death rate of 670 adult patients with COVID-19 and cancer (14.93%) was higher compared to that of 14,840 adult patients with COVID-19 without cancer (5.26%) and compared to that of 270,380 adult and senior patients with cancer but no COVID-19 (4.03%). Hospitalization rate of patients with a recent cancer diagnosis and COVID-19 (47.76%) was higher compared to that of patients with COVID-19 but no recent cancer diagnosis (4.26%) (*p* < 0.001) and higher compared to that of patients with recent cancer diagnosis but no COVID-19 (12.39%) (*p* < 0.001). Overall, COVID-19 and cancer had a synergistic effect on death rate and on hospitalization rate. In this analysis, patients with TC were at significantly increased risk for COVID-19 infection (adjusted odds ratio (aOR), 3.94; 95% CI, 2.88–5.40). The odds ratio of COVID-19 infection for patients with TC decreased after adjusting for COVID-19 risk factors, indicating that these factors contributed to the risk for COVID-19 infections (aOR, 3.10; 95% CI, 2.47–3.87; *p* < 0.001). No analysis of outcomes according to specific cancer was conducted in this study [85].

Cancer patients are considered at increased risk for getting infections due to compromised immune system. Other factors, such as socio-economic status and behavioral and lifestyle factors, may also increase the risk of COVID-19 infection in cancer patients. However, due to limited relevant information in most databases, evaluation of the impact of these factors on the risk of COVID-19 infection among patients with cancer is not often feasible [85]. 

The outcome of COVID-19 in patients with TC was evaluated in a nationwide retrospective study leveraging the Turkish Ministry of Health database. The authors compared a cohort of 388 COVID-19 patients with TC to 388 gender-matched COVID-19 patients without TC from 11 March to 30 May 2020 in terms of mortality and morbidity. This study showed that the mortality ratio was similar in the TC and the non-TC group. In the TC group, factors correlated with mortality in univariate analysis were age (*p* < 0.001), DM 2 (*p* = 0.016), asthma/chronic obstructive pulmonary disease (COPD) (*p* = 0.041), heart failure (*p* = 0.021), chronic kidney disease (*p* < 0.001), prior coronary artery disease (*p* = 0.039), RAS blocker (*p* = 0.036), and lymphopenia (*p* < 0.001). No impact of RAI treatment and cumulative RAI dose on the severity and mortality of COVID-19 was observed; however, TC diagnosis was an independent risk factor of hospitalization (OR, 0.38; 95% CI, 0.27–0.54; *p* < 0.001). Additional independent risk factors of hospitalization were age (OR, 1.03; 95% CI, 1.01–1.05; *p* = 0.001), and positivity of CT findings of COVID-19 (OR, 3.14; 95% CI, 2.08–4.76; *p* < 0.001) [88]. 

Kathuria-Prakash et al. analyzed the COVID-19 outcomes in relation to demographic, DTC, and treatment data in a retrospective cohort study of patients with DTC and COVID-19 from 2 academic Los Angeles healthcare systems. Among 21 patients with DTC and COVID-19, the incidence of hospitalization was 38.1%, and the incidence of COVID-19 related death was 9.5%, the latter being comparable to that of patients with non-thyroid malignancy (7.6%; *p* = non-significant). No parameter related to DTC (i.e., primary tumor size, risk of recurrence according to ATA classification, DTC status, and response to therapy near the time of COVID-19 diagnosis) was significantly associated with the severity of COVID-19 in DTC patients. Notably, administration of a cumulative RAI dose of ≥100 mCi led to higher rate of hospitalization for COVID-19 compared to no administration of RAI or administration of lower RAI doses, but this association did not reach statistical significance. Evaluation of non-DTC clinical parameters recognized as risk factors for unfavorable COVID-19 outcomes, including older age, male sex, and medical comorbidities, showed that older age and one comorbidity other than DTC were significantly associated with COVID-19 hospitalization (*p* = 0.047 and *p* = 0.024, respectively). Among patients with DTC, hospitalization related to COVID-19 was more common in patients with DM 2, lung disease, or cardiovascular disease [89]. 

Overall, current data on the impact of (D)TC on COVID-19 severity are limited and show no impact of (D)TC on COVID-19-related mortality, contrary to the designation of cancer as prognostic factor for unfavorable outcome of COVID-19 [10]. However, current data in terms of the impact of DTC on COVID-19 hospitalization are conflicting. No explanation for these discordant and inconclusive data exists so far. Table 5 lists the varying, occasionally discordant data on the impact of cancer on COVID-19 severity between cancer patients in general and (D)TC patients.

From a critical viewpoint, data that show the absence of a detrimental impact of (D)TC on COVID-19 outcome, contrary to the designation of other cancers as prognostic factor for unfavorable outcome of COVID-19, may be attributed to the indolent nature and the favorable prognosis of most cases of PTC—the most common histologic type of TC.

### Are Obese Patients with (D)TC at Increased Risk for Unfavorable COVID-19 Outcomes?

Interestingly, in the retrospective cohort study of 21 patients with DTC and COVID-19 from two academic Los Angeles healthcare systems conducted by Kathuria-Prakash et al., hospitalization was required for more than half of patients (4/7, 57.1%) with a BMI > 30 kg/m^2^ as opposed to only 28.6% (4/14) of patients with a lower BMI [89]. This finding is in consistence with the association of obesity with increased COVID-19 severity in non-cancer COVID-19 patients compared to no obesity [12,13], but contradicts the constantly reported absence of impact of obesity on COVID-19 severity in cancer patients [49,50,51]. Nevertheless, the small sample size of the study of Kathuria-Prakash et al. hampers the generalization of this finding, indicating the need for further research. From a critical viewpoint, the indolent nature, and the favorable prognosis of most cases of PTC—the commonest histologic type of TC—may account for the absence of a postulated protective effect of obesity on COVID-19 outcome for TC patients, contrary to what is reported for other tumors.

## 7. The Hypothesis of the Oncogenic Potential of SARS-CoV-2

The hypothesis of the oncogenic potential of SARS-CoV-2 is discussed herein as the rationale behind the pending question of whether SARS-CoV-2 can cause (D)TC.

Confronted with “long-haul COVID-19”, COVID-19 survivors are increasingly reported to experience a constellation of clinical symptoms that synthesize the post-acute COVID-19 syndromes, an issue beyond the scope of the present review [90,91,92]. Herein, we focus on the alarming hypothesis of post-acute COVID-19 increased risk of development of malignant neoplasms [90].

The rationale for this hypothesis is the identification of a causative link between viral infections and cancer, which dates back to the advent of 20th century [93,94]. So far, advances in research on cancer virology have indicated certain viruses as a causative factor of 12–20% of cancer worldwide, mainly via harnessing host’s proteins, hijacking proliferating human cells, and triggering genetic and epigenetic alterations. Seven viruses have been causally associated with human oncogenesis: Epstein Barr virus (EBV), high-risk human papillomaviruses (HPV16/18), hepatitis B and C viruses (HBV, HCV respectively), human T-cell lymphotropic virus-1 (HTLV-1), Kaposi’s sarcoma herpesvirus (KSHV), and Merkel cell polyomavirus [95].

The criteria for official assignment of causality between virus and cancer are long-term consistency of association between virus and cancer at the epidemiological and/or molecular level, and demonstration of the tumorigenicity of the virus in animal models, or demonstration of the transforming ability of the virus in cell culture [96].

Whether SARS-CoV-2 has an oncogenic potential can be answered only after years of epidemiological surveillance [97], and concomitant verification of two conditions: persistence of infection in the host, and identification of a pathogenetic link between oncogenesis and infection.

The persistence of SARS-CoV-2 is a new paradigm of COVID-19, yet not confirmed. Some indicative data are: (i) persistent shedding of viral genetic material long after recovery of the acute disease accompanied by a negative RT-PCR test result for SARS-CoV-2, raising concern about re-infection or reactivation of latent infection; (ii) tropism of SARS-CoV-2 towards endothelium, which may foster its persistence in tissues other than tissues associated with the known symptoms of COVID-19; (iii) involvement of central nervous system in the COVID-19, as indicated by positivity of RT-PCR in cerebrospinal fluid, a site with a known immune privilege, which could provide protection to SARS-CoV-2 and foster its latency [98,99].

As regards the mechanisms underlying the potential oncogenic effect of SARS-CoV-2, little evidence exists so far. A relevant speculative mechanism has been suggested by Stingi et al. [99], according to which SARS-CoV-2 counteracts the tumor suppressor proteins pRb and p53 [100,101,102,103]. This hypothesis is based on data derived from SARS-CoV-1. The interaction between the non-structural protein (Nsp) 15 (Nsp 15) of SARS-CoV-1 and the pRB of infected cells via a LXCXE motif has been shown to induce the nuclear export and ubiquitination of pRB, allowing its proteasomal degradation. Additionally, the Nsp3 protein of SARS-CoV-1 has been shown to stabilize the E3 ubiquitin ligase ring-finger and CHY zinc-finger domain-containing 1 (RCHY1), promoting the degradation of p53, and decreasing the levels thereof. Similar effects may be exerted by the Nsp15 and Nsp3 nucleases of the SARS-CoV-2 genome, which share, respectively, 88.7% and 76% sequence similarity with their counterparts of the SARS-CoV1 genome [100].

On the other hand, an argument against the oncogenic effect of SARS-CoV-2 is its cytotoxic effect, which reduces the chances for cell transformation. Although some cells survive following SARS-CoV-2 infection, a decreased possibility of cell transformation is expected due to virus-induced cell cycle arrest and activation of the cascade of apoptosis. However, whether cell transformation could occur in certain infected cell types in case of abortive cycles of the virus and of diminished cytopathic effects of virus remains unknown [100].

To explore a potential direct mutagenic effect of SARS-CoV-2, further in vitro and animal studies are needed; however, interpretation of relevant results will be daunting due to the genotoxic effect of sustained immune/inflammatory response per se. A potential synergistic oncogenic effect between SARS-CoV-2 and obesity is insinuated by the in vitro ability of the downregulation of p53 protein to mediate the obesity-driven tumor progression attributed to increased levels of C1q/TNF-related protein 1 (CTRP1), an adiponectin paralog [103].

## 8. Future Perspectives

To ensure the continuity of (D)TC care amid the rapidly evolving, multifaceted, challenges posed by the COVID-19 pandemic, concerted efforts should be directed towards filling current knowledge gaps in the COVID-19-DTC association and informing the decision-making.

Primarily, an interesting future perspective is to devise new mechanism-guided, tailored, therapeutic and preventive strategies for the management of (D)TC in these demanding times and beyond. To this end, more research is needed to understand and harness the pathophysiological landscape of the COVID-19–(D)TC association. In that respect, leveraging huge high-throughput “multi-omics” datasets could untangle the molecular basis of the interplay between inflammation, immunity, and obesity that governs the COVID-19–(D)TC association. Such information may lead to validation of patient-specific and cancer-specific biomarkers of this association and development of dual targeted treatments.

Especially, improved understanding of the immune-inflammatory mechanisms underlying the COVID-19–(D)TC association may provide the rationale for the development of novel treatments or the repurposing of existing treatments to combat concomitantly COVID-19 and cancer. For instance, a phase II expanded access trial is ongoing evaluating tocilizumab—a recombinant humanized anti-interleukin-6 receptor (IL-6R) monoclonal antibody—in cancer patients with COVID-19 [104]. The rationale of this trial is that preliminary data show efficacy of tocilizumab as anticancer agent [105,106] and as agent to reduce the risk of mechanical ventilation in hospitalized COVID-19 patients [107]. Furthermore, the dual effects of certain phosphodiesterase (PDE) inhibitors, anti-inflammatory [108] and anticancer [109,110], merit further evaluation in the setting of the COVID-19–(D)TC association.

Given the presumed role of the oxidative stress in the molecular background of the COVID-19-cancer association, it would be of interest to investigate a potential anticancer efficacy of antioxidant therapies that are currently under evaluation as a strategy to reduce the severity of COVID-19 [111]. A pitfall to this perspective is the differential regulation of pro-oxidant and antioxidant systems according to cancer stage. ROS promote cancer initiation at early precancerous and neoplastic stages. At advanced cancer stages, ROS promote the apoptosis of cancer cells, but cancer cells hijack antioxidant systems to evade apoptosis. Consequently, antioxidant therapies may be beneficial for the host only at initial stages of cancer [112].

Deciphering the role of obesity in the molecular background of the COVID-19–(D)TC association should be prioritized considering its modifiable nature. A barrier to this perspective is the significant heterogeneity of the metabolic profile of obese individuals, integrating genetic, epigenetic, socio-economic, and environmental factors [11]. Establishment of a universal terminology of distinct subgroups of obese patients, and validation of corresponding classification markers and indices is mandatory to identify differences between metabolically healthy and metabolically unhealthy obese individuals [113,114,115]. To combat the growing rates of obesity worldwide, nominated as “globesity” [116], medical authorities and policy makers need to intensify their coordinated actions to implement integrated and intersectoral health strategies [117,118]. Racial and ethnic disparities influencing not only the incidence of obesity, and of (D)TC, but also the severity of COVID-19 [119,120] should be considered.

Another promising area of future research is to evaluate the impact of (D)TC, especially of distinct patient-specific and cancer-specific factors, on COVID-19 outcome. Several issues concerning the impact of DTC treatment on COVID-19 outcome await clarification. First, a cautious patient selection for RAI treatment might be essential in the COVID-19 era due to the potential RAI-driven attenuation of immune responses in parallel with the concern about the safety/efficacy balance of RAI for most intermediate-risk and low-risk subgroups of (D)TC [121,122,123]. Second, whether L-T4 binding to integrin αvβ3 can diminish the integrin αvβ3-mediated internalization of the SARS-CoV-2 is yet to be investigated. Third, as kinases are involved in the virus-induced cytokine storm, tyrosine kinases inhibitors (TKI)—used for treatment of advanced (D)TC—are under evaluation as antiviral agents against SARS-CoV-2 [124,125]. Fourth, L-T4 titration after (D)TC surgery in patients with COVID-19 may be hampered by TSH suppression due to non-thyroidal illness syndrome (NTIS) caused by COVID-19, or due to exogenous corticosteroids used for treatment of severe COVID-19 [126].

Given the increasing recognition of subacute thyroiditis (SAT) as manifestation of COVID-19 [127,128,129,130] and the higher than initially presumed prevalence of PTC in SAT patients (4.4%), COVID-19 pandemic may provide the opportunity to revisit the SAT-PTC association [131].

To counteract the pitfalls that are intrinsic in the “sci-infodemic” (i.e., the publishing pandemic) during the COVID-19 pandemic [132], several methodological issues concerning COVID-19 research need to be further addressed, namely (i) sampling errors resulting in non-representative sample, producing non-generalizable results; (ii) variety of diagnostic criteria of COVID-19; (iii) heterogeneity of inclusion criteria of COVID-19 patients between studies; (iv) incomplete reporting of COVID-19 outcomes hampering subgroup analyses; (v) lack of standardized definitions of COVID-19 outcomes; (vi) inadequate follow-up.

Future health strategies for the management of (D)TC should be customized according to different pandemic phases. Additionally, more data are required to determine the impact of telemedicine on (D)TC management [133].

From an holistic viewpoint, COVID-19 pandemic has incited a novel social-economic and cultural landscape, causing a lot of distress due to altered lifestyle, generalized fear, pervasive community anxiety, and financial consequences [134]. In this psychosocial context, specialized health professionals, telephone helplines, active social networks, and dedicated forums are required to support the psychologically vulnerable individuals to deal with (D)TC diagnosis and treatment [135]. Finally, thyroid is interrelated with the psychological, mental, and physiological principals of our being, all threatened by the novel socio-economic and cultural landscape. It is presumed that the coming years will mandate more than ever an holistic approach to thyroid diseases, including (D)TC, because the psychosocial aspects of our being have already been correlated with thyroid function through research in a new realm of interest called Psychoneuroendocrinology. Psychoneuroendocrinology—a complex blend of psychiatry, psychology, neurology, biochemistry, and endocrinology—addresses, among others things, the psychobiological factors that influence the thyroid function in the context of the response to stress. Thus far, hypothalamus–pituitary–thyroid (HPT) axis dysfunction has been associated with major depression [136], post-traumatic stress disorder (PTSD) [137], anxiety disorders [138,139], and suicide risk related to major depressive disorder [140]. Critical life events often precede the onset of autoimmune thyroid diseases, and exposure to psychosocial stress triggers an immediate activation of HPT axis [141]. The activity of TRH neurons is inhibited by negative energy balance situations such as fasting, inflammation or chronic stress [142]. Indeed, very recently, Keestra et al. suggested an evolutionary ecology framework according to which HPT axis is considered a dynamic, adaptive system with a key role in “mediating life-history trade-offs between the functions of reproduction, growth, immunity and basal metabolic rate” [143] in the setting of demanding situations, including depression, psychosocial stress, and traumatic stress [144,145]. The absence of a causality between the psychosocial stress and the thyroid disorders in the setting of COVID-19 pandemic does not undermine the magnitude of an holistic approach to this issue, which will enable an integrated patient-oriented management of thyroid disorders, including DTC, during the challenging COVID-19 pandemic and beyond.

**Figure 4:**
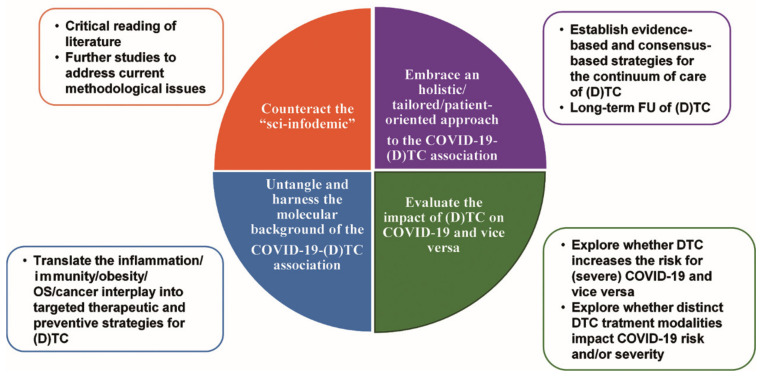
The main future perspectives in understanding and harnessing the COVID-19–(D)TC association. Abbreviations: COVID-19, coronavirus disease 19; (D)TC, (differentiated) thyroid cancer; FU, follow-up.

Figure 4 illustrates the main future perspectives in understanding and harnessing the COVID-19–(D)TC association.

## 9. Conclusions

In response to call for caution regarding the COVID-19–(D)TC association, the scientific world acknowledges that there is still more to learn about the underlying interplay between obesity, inflammation, immunity, and cancer. Capitalizing on the COVID-19–(D)TC association may not be far away provided that improved understanding of the molecular background thereof will be translated into therapeutic and preventive strategies. Meanwhile, an evidence-based, risk-stratified, and consensus-based decision-making is embraced to provide consistent, safe, and high-quality care for (D)TC patients. From an optimistic standpoint, the “silver lining” in the dark cloud of COVID-19 is to leverage it as a catalyst for innovative patient-oriented health strategies integrating new knowledge with breakthrough technologies. A hopeful path forward is to entwine cancer care with the COVID-19 care in the setting of strengthened health systems.

## Figures and Tables

**Table 1:** Controversial data on the association between obesity and (D)TC.

Controversy over the Association of Obesity with (D)TC	
Data Sustaining the Association of Obesity with (D)TC [Ref].	Data Sustaining no Association of Obesity with (D)TC [Ref].
➢ TC is included among the 13 cancer types causally associated with obesity, according to the landmark report of the International Agency for Research on Cancer (IARC) [25]. ➢ 1.8% of TC-related deaths in females worldwide is attributable to high BMI, a percentage higher compared to males [18]. ➢ Compared to normal weight (18.5–24.9 kg/m^2^), overweight (25.0–29.0 kg/m^2^) and obesity (≥30.0 kg/m^2^) increased: PTC risk by 1.26-fold and 1.30-fold respectively risk of large (>4 cm) PTC (HR, 2.93 and 5.42 for overweight and obesity respectively) [22]. ➢ During 1995–2015: Increase of PAF for overweight and obesity: from 11.4% to 16.2% for all PTC and from 51.4% to 63.2% for large PTC. Overweight or obesity were responsible for 13.6% and 57.8% of the annual percent changes in all and large PTC incidence, respectively [22]. ➢ The association of obesity with increased TC risk is mediated by mechanisms implicating adipokines and chronic inflammation [31]. ➢ Compared to normal-weight and overweight, obese individuals show statistically significant increased TC risk: 25% and 55%, respectively [21]. ➢ Association of each 5-unit increase in BMI, 5 kg increase in weight, 5 cm increase in W or H circumference and 0.1-unit increase in W/H ratio with 30%, 5%, 5%, and 14% increased TC risk, respectively [21]. ➢ Significant positive association of obesity with PTC, FTC, and anaplastic TC [21]. ➢ Association of both general and abdominal adiposity with TC [21]. ➢ Association of obesity with a significantly increased TC risk (adjusted RR, 1.33) [24]. ➢ Five key players of the link between excessive weight and TC: thyroid hormones, insulin resistance, adipokines, inflammation, and sexual hormones [28].	➢ No association of obesity with DTC [37]. ➢ No association of BMI with TC [38]. ➢ Positive association of higher BMI with benign thyroid nodular disease (OR, 1.15; 95% CI, 1.08–1.22), higher W/H (OR, 1.16; 95% CI, 1.09–1.23), but not with TC [39]. ➢ Mendelian randomization: no causal association between obesity and benign nodular thyroid disease or TC [39].

Abbreviations: BMI, body mass index; FTC, follicular thyroid carcinoma; H, hip; HR, hazard ratio; PAF, population attributable fractions, PTC, papillary thyroid carcinoma; RR, relative risk; TC, thyroid cancer; Ref, reference, W, waist.

**Table 2:** Discord in the contribution of obesity to COVID-19 outcome in non-cancer and cancer patients.

Discordance about the Contribution of Obesity to Unfavorable COVID-19 Outcome in Non-Cancer and Cancer Patients	
Obesity is Related to Unfavorable COVID-19 Outcome in Non-Cancer Patients	Obesity is Not Related to Unfavorable COVID-19 Outcome in Cancer Patients
➢ Significant association of obesity with increased risk of mortality among patients with COVID-19 (RR_adjust_: 1.42 (95%CI, 1.24–1.63, *p* < 0.001) [13]. ➢ Inflammatory and immune mechanisms may increase susceptibility of obese individuals to severe COVID-19 and poor COVID-19 clinical outcome [43]. ➢ Inflammation, RAS activation, elevated adipokines and higher ectopic fat may contribute to COVID-19 severity in obese individuals [44]. ➢ Obesity is associated with increased risk of severe COVID-19. Limited data exist on metabolic parameters (such as BMI and levels of glucose and insulin) in patients with COVID-19 [45]. ➢ In general patient population with COVID-19, obesity is a determinant of increased morbidity and mortality [46]. ➢ Metabolic impairments and chronic low-grade systemic inflammation related to obesity create a pro-inflammatory microenvironment, building on SARS-CoV-2 related cytokine storm [47]. ➢ Patients with severe COVID-19 have a higher BMI than patients with no severe COVID-19 (WMD, 2.67; 95% CI, 1.52–3.82) [48]. ➢ COVID-19 patients with obesity have a worse outcome than those without obesity (OR = 2.31; 95% CI, 1.3–4.12) [48].	➢ Obesity is not a determinant of increased COVID-19 related morbidity and mortality in cancer patients [46]. ➢ Obesity is not a risk factor for COVID-19 related clinical worsening and overall survival (HR, 1.16; 95% CI, 0.36–3.69) in cancer patients a univariable Cox proportional model [49]. ➢ No association of obesity with increased 30-day mortality in cancer patients with COVID-19 (OR, 0.84; 95% CI, 0.50–1.41), not even after adjusting for age, sex, and smoking status (OR, 0.99; 95% CI, 0.58–1.71) [50]. ➢ No correlation of obesity with mortality (OR, 0.95, 95% CI, 0.74–1.23) in cancer patients with COVID-19 [51].

Abbreviations: BMI, body mass index; CI, confidence interval; COVID-19, coronavirus disease 2019; HR, hazard ratio; OR, odds ratio; RAS, renin-angiotensin-aldosterone system; Ref, reference; RR_adjust_, relative risk adjusted; WMD, weighted mean difference.

**Table 3:** COVID-19 pandemic guidelines for management of thyroid tumors with focus on DTC by diverse expert committees.

Committee of 67 experts (45 endocrine surgeons and 22 endocrinologists) from multiple countries ^a^ [73].	
FNA result	Recommendation
➢ Any of the following: Atypia or follicular lesion of undetermined significance (Bethesda 3) repeated twice. Hürthle cell neoplasia or follicular neoplasia (Bethesda 4). Papillary thyroid microcarcinoma (<1 cm).	➢ Alternative options: Postponement of surgery for 3–6 months and follow-up with active surveillance until the end of the pandemic. OR Treatment with minimally invasive ablation techniques including laser, microwave, or radiofrequency ablation.
➢ Papillary thyroid carcinoma without LNM in the neck.	➢ Alternative options: Postponement of surgery for 3–6 months OR Treatment with minimally invasive ablation techniques including laser, microwave, or radiofrequency ablation.
➢ Papillary carcinoma with LNM at central or lateral neck.	➢ Postponement of surgery for 3–6 months.
Committee of French-speaking Association of Endocrine Surgery (AFCE) [74].	
FNA result	Recommendation
➢ DTC ≥ 2 cm with or without LNM without signs of locoregional aggressiveness.	➢ Postponement of surgery until the end of crisis, but, thereafter, surgery should be prioritized with a delay <3 months.
➢ DTC < 2 cm with or without LNM without signs of locoregional aggressiveness.	➢ Surgery can be performed long after the end of pandemic.
Committee of Turkish Association of Endocrine Surgery [75].	
FNA result	Recommendation
➢ Indeterminate nodules (Bethesda 3 and 4).	➢ Follow-up. AND ➢ BRAF or TERT positivity does not impose immediate surgery.
➢ Any of the following: DTC with ATA high risk of recurrence (i.e., large tumors, or tumors with gross extrathyroid extension, or tumors with <3 LNM or any LNM <3 cm, or tumors with distant metastasis) Rapid growth of tumor	➢ Immediate surgery
➢ DTC with ATA low and intermediate risk of recurrence (i.e., DTC without compressive symptoms and signs, apparent LNM, or voice changes).	➢ Postponement of surgery for 3–6 months. AND ➢ In case of extended observation period, US repeated three months after initial diagnosis may allow further delay of surgery.
➢ Intrathyroidal papillary microcarcinoma	➢ Observation until end of pandemic. Certain patients can remain under active surveillance with repeated US even after end of pandemic.
Committee of Brazilian Society of Endocrinology and Metabolism (SBEM) [76].	
FNA result	Recommendation
➢ TC with low risk of recurrence.	➢ “Surgical procedures could and should be postponed safely during the pandemic period.” AND ➢ Safe postponement of RAI.

^a^ Austria, Belgium, Bulgaria, Greece, Italy, the Netherlands, Poland, Russia, South Korea, Spain, Sweden, Switzerland, Turkey, Ukraine, the United Kingdom and USA. Abbreviations: ATA, American thyroid association; DTC, differentiated thyroid carcinoma; FNA, fine needle aspiration; LNM, lymph node metastases; RAI, radioiodine; TC, thyroid cancer; US, ultrasound.

**Table 4:** The challenges in the oncological strategy for DTC due to COVID-19 pandemic.

Challenges in in the Oncological Strategy for DTC Due to COVID-19	
Challenge I: Appropriate patient selection for FNA	
Study type [Ref]	Results
Analysis of thyroid FNA trends at Federico II University of Naples before, during and after first lockdown in Italy ^a^. [69]	➢ Decrease of AWN of weekly FNA from 62.1 to 23. ➢ Weekly proportion of benign diagnoses: decreased by 12% ➢ Weekly proportion of high-risk diagnoses: increased by 6%.
International survey of cytopathologic laboratories in 23 countries. [70]	➢ Decrease of percentage of thyroid samples collected during 4 weeks of COVID-19 lockdown versus the corresponding period of 2019: 3.26% versus 5.02% (*p* < 0.001). ➢ Less profound overall decrease in thyroid FNA cytology volume for samples with higher rate of malignancy.
Analysis of databases of China, South Korea, Iran, and Italy concerning thyroid surgery during 3 COVID-19 pandemic phases ^b^. [71]	➢ Decreased overall collected sample volume during 4 weeks of COVID-19 lockdown versus the corresponding period of 2019: 104,319 samples versus 190,225 samples. ➢ Reduction in outpatient FNA by: ➢ 99.7% (phase I) ➢ 62.9% (phase II) ➢ 30.1% (phase III)
Challenge II: Reschedule of DTC surgery	
Study type [Ref]	Results
Analysis of databases of China, South Korea, Iran, and Italy concerning thyroid surgery during 3 COVID-19 pandemic phases ^b^. [71]	➢ Reduction of thyroid surgeries versus 2019: ➢ Phase I: No thyroid surgery. ➢ Phase II: reduction in surgery of advanced DTC (mainly of stage T1b N1a ^c^). ➢ Phase III: reduction in surgery of advanced DTC (mainly of T3bN1b ^d^). ➢ Reduction of mean surgical time versus 2019: ➢ to 58.3 ± 11.26 min in phase II (*p =* 0.000). ➢ to 55.3 ± 13.32 min in phase III (*p =* 0.000). ➢ Reduction of postoperative hospitalization versus 2019: ➢ to 2.8 ± 0.9 days in phase II (*p =* 0.000). ➢ to 3.3 ± 1.0 days in phase III (*p =* 0.008).
Challenge III: Assessment of delays and disruptions in DTC care	
Study type [Ref]	Results
Systematic review of 62 studies. [77]	➢ Head and neck cancer patients: 11% of total patients. ➢ Interruptions in patient care occurred in facilities (up to 77.5%), supply chain (up to 79%), and availability (up to 60%). ➢ Reduction in routine activity of cancer services, and number of cancer surgeries. ➢ Delay in radiotherapy. ➢ Delay, reschedule, or cancellation of outpatient visits.
Study on PTC treatment plans of 12 PTC patients. [78]	➢ No delay of planned PTC surgery. ➢ Decreased availability of RAI due to lockdown across international borders. ➢ Replacement of the usual method for the delivery of RAI after L-T4 withdrawal with the rh-TSH-stimulated method in most patients. ➢ Distress for patients who could not afford the rh-TSH-stimulated method.
Challenge IV: The safety of DTC surgery in the COVID-19 era.	
Study type [Ref]	Results
Systematic review on thyroid surgery. [80]	➢ Cross-infections in 14 patients (1.9%). ➢ Severe pulmonary complications of COVID-19 in 0.4% of infected patients. ➢ Complications related to surgery (data from 4 studies): 20.2% of patients (mainly hypoparathyroidism and recurrent laryngeal nerve injury).
Analysis of databases of China, South Korea, Iran, and Italy concerning thyroid surgery during 3 COVID-19 pandemic phases. [71]	➢ No cases of COVID-19 related complications during the perioperative period in phases I, II, and III of lockdown in China, South Korea, Iran, and Italy.
International, observational cohort study of 1137 consecutive head and neck cancer patients subjected to primary surgery in 26 countries. [81]	➢ Thyroid: 2nd most common site of surgery (21%). ➢ Overall 30-day mortality after TC surgery: 1.2% (similar to pre-COVID data). ➢ Incidence of positivity of SARS-CoV-2 tests within 30 days of surgery in patients: 3%. ➢ Incidence of positivity of SARS-CoV-2 tests within 30 days of surgery in the surgical team: 3%. ➢ Severe respiratory complications in 44.8% of infected patients. ➢ Medical and surgical complications within normal ranges. ➢ Significant association of advanced tumor stage with admission to critical care.

^a^ before: 1 January 2019 to 13 March 2020; during: 14 March to 15 May; after: 16 May to 7 July first lockdown in Italy. ^b^ phase I (25 January–25 February 2020); phase II (26 February–19 March); phase III (20 March–20 April). ^c^ T1b N1a: the tumor is between 1 cm and 2 cm completely inside the thyroid (T1b) and has spread to lymph nodes close to the thyroid in the neck pretracheal, paratracheal, and prelaryngeal lymph nodes) (level VI), or in the upper chest (the superior mediastinal nodes) (level VII) (N1a). ^d^ T3bN1b: the tumor is any size and has grown into one or more of the muscles beside the thyroid (strap muscles) (T3b), and to lateral lymph nodes (levels I, II, III, IV, and V) or to lymph nodes behind the throat (retropharyngeal) (N1b). Abbreviations: AWN, average weekly number; COVID-19, coronavirus disease 2019; DTC, differentiated thyroid cancer; FNA, fine needle aspiration; PTC, papillary thyroid carcinoma; RAI, radioiodine; Ref, references; rh-TSH, recombinant human thyroid-stimulating hormone.

**Table 5:** Varying, occasionally discordant, data on the impact of cancer on COVID-19 severity between cancer patients in general and (D)TC patients.

Impact of Cancer in General on COVID-19 Severity	Impact of (D)TC on COVID-19 Severity
➢ Cancer —especially the active one—was indicated as independent factor associated with increased 30-day mortality in COVID-19 patients in logistic regression analysis, after partial adjustment [50]. ➢ COVID-19 related mortality rate in cancer patients was 28.6%, more than ten times higher than that reported in all COVID-19 patients [82]. ➢ Patients with solid or hematological malignancies and SARS-CoV-2 infection have a high probability of mortality [83]. ➢ COVID-19 patients with cancer had higher risks in terms of all severe outcomes [84]. ➢ Patients with COVID-19 and cancer versus patients with COVID-19 without cancer versus patients with cancer but no COVID-19 (4.03%): Mortality: 14.93% versus 5.26% versus 4.03% Hospitalization: 47.76% versus 4.26% versus 12.39% [85]. ➢ Metastatic lung cancer and hematological cancer confer the greatest risk of severe complications of COVID-19 and related mortality [87].	➢ Similar COVID-19 related mortality in the TC and the non-TC group [88]. ➢ TC is an independent risk factor of COVID-19 related hospitalization (OR, 0.38; 95% CI, 0.27–0.54; *p* <0.001) [88]. ➢ In DTC patients, the incidence of COVID-19 related hospitalization was 38.1%, and the incidence of COVID-19 related death was 9.5%, the latter being comparable to that of patients with nonthyroid malignancy (7.6% *p* = non-significant) [89].

Abbreviations: CI, confidence interval; COVID-19, coronavirus disease 2019; (D)TC, differentiated thyroid cancer; OR, odds ratio; Ref, reference.
